# Dupuytren's Contracture

**Published:** 2013-01-15

**Authors:** Aditya Sood, Angie Paik, Edward Lee

**Affiliations:** Division of Plastic Surgery, University of Medicine and Dentistry of New Jersey, Newark, NJ

**Figure:**
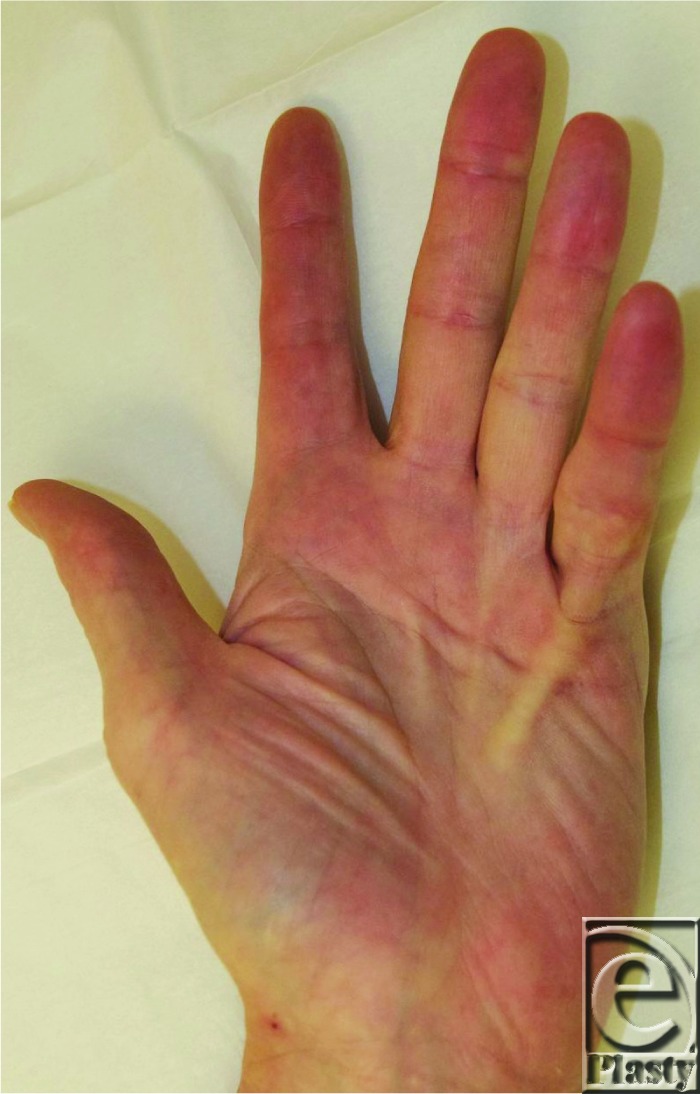


## DESCRIPTION

Patient is a 65-year-old right-hand-dominant man. He presents with a 15-year history of progressive left small finger contracture with occasional palmar pain. On examination, he has a palpable palmar cord. Contraction of left small finger metacarpophalangeal (MP) joint was noted to be 60°.

## QUESTIONS

1. **What is Dupuytren's disease?**
2. **What is the pathophysiology?**
3. **What are the indications for intervention?**
4. **What treatment options are available?**

## DISCUSSION

Dupuytren's disease is a fibroproliferative disorder of the palmar fascia. It is characterized by hyperproliferation of type 3 collagen scar tissue in the palms and digits.^6^ This results in nodules or cords that can progress to contraction at the MP and proximal interphalangeal (PIP) joints with hyperextension of the distal interphalangeal joints. While Dupuytren's disease has no single etiology, a genetic predisposition has been identified. It is thought to be autosomal dominant with variable penetrance.^2^ It is most often found in older men of northern European descent. Common associations include alcohol, smoking, manual labor, diabetes, and epilepsy.

Histologically, the disease has 3 stages: proliferative, involution, and residual phases. During the proliferative stage, mediators such as transforming growth factor β1 induce cell transformation to myofibroblasts. During the involution stage, myofibroblasts produce collagen and alpha-smooth muscle actin, which are thought to play a role in contraction. These 2 elements create the nodules and contraction of the disease. Finally, during the residual phase, the nodules regress and leave behind thickened cords. Other mediators of the disease process that have been looked at include fibroblast growth factor, platelet-derived growth factor, myoglobin, tyrosine kinase, and metalloproteinase.^6^

Dupuytren's disease manifests first in the skin with thickening and tenderness over the affected area. The disease can progress to nodule formation and/or cord formation that may lead to a decreased range of motion. Over time, flexion contracture of the MP or PIP joints may develop. In addition to decreased range of motion, the fibrous nodules and cords can lead to nerve compression and vascular displacement.

A simple clinical test, called the tabletop test, can determine if a contracture is present. If one can lay the hand palm down flat on a table, a contracture is not present. Surgery is recommended when any of the following conditions are present: (1) MP contractures of more than 30° or (2) PIP involvement with functional impairment or neurovascular compromise.

Treatment includes both surgical and nonsurgical options. Surgery remains the mainstay of treatment. Surgical options include needle aponeurotomy, open or percutaneous fasciotomy, and open fasciectomy. The most widely used contracture release is a partial fasciectomy in which only the diseased fascia is excised. Incisions include transverse, longitudinal with Z-plasties, Bruner-type zig-zag incisions, and multiple Y-V advancement flaps. After surgery, the hand is splinted with the joints fixed in extension followed by a course of hand therapy.

Until fairly recently, nonoperative treatments including vitamin E and steroids have been unsuccessful.^2^ In 2010, the Food and Drug Administration approved Xiaflex (Auxilium Pharmaceuticals, Malvern, PA) (collagenase clostridium histolyticum) as a nonsurgical treatment for adults with Dupuytren's contracture with palpable cord. Xiaflex has shown promising early results.^3^

The treatment of Dupuytren's disease is fraught with concern. The patient's symptoms and complaints are often mild compared to the risks of intervention—tendon rupture and neurovascular injury. In addition, there is a high recurrence rate whether treated surgically or nonoperatively.^4^

**Figure 1:**
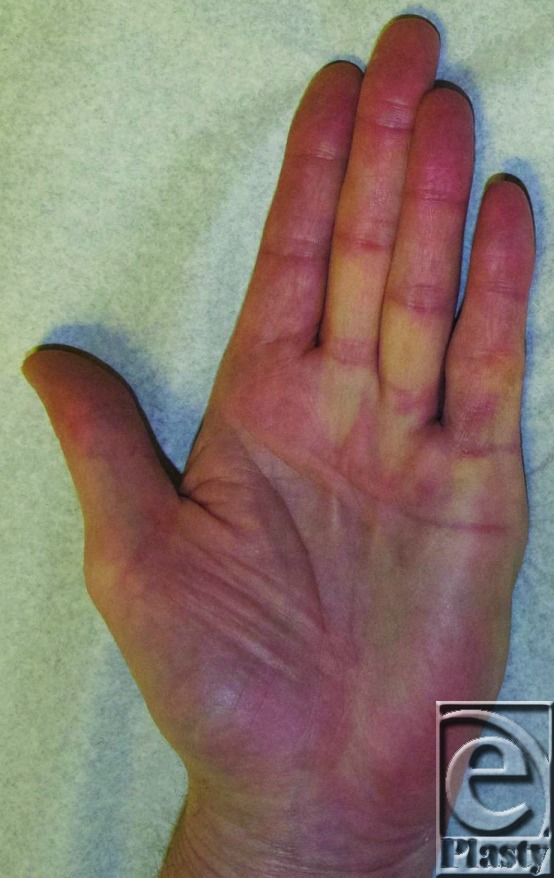
Two weeks postoperative. The patient was able to fully hand extend fifth MP joint and lay hand flat on table.

**Figure 2:**
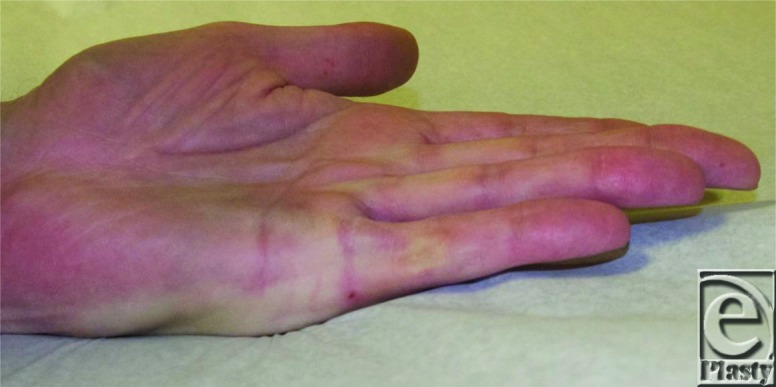
Two weeks postoperative. Patient was able to fully extend all digits of the affected hand.

In the case described earlier, the patient opted for nonsurgical therapy consisting of Xiaflex injection followed by finger stretching and ulnar gutter splint. Two weeks after operation, patient displayed 5° of flexion contracture in left fifth MP joint, excellent range of motion, no pain, and he was very happy with the results (Figs 1 and 2).
